# Are the processes recommended by the NHMRC for improving Cardiac Rehabilitation (CR) for Aboriginal and Torres Strait Islander people being implemented?: an assessment of CR Services across Western Australia

**DOI:** 10.1186/1743-8462-6-29

**Published:** 2009-12-30

**Authors:** Sandra C Thompson, Michelle L DiGiacomo, Julie S Smith, Kate P Taylor, Lyn Dimer, Mohammed Ali, Marianne M Wood, Timothy G Leahy, Patricia M Davidson

**Affiliations:** 1 Centre for International Health, Curtin University of Technology, Bentley Campus, Perth, Western Australia 6102, Australia; 2 Curtin Health Innovation Research Institute (CHIRI), Curtin University, GPO Box U1987 Perth, Western Austrlaia 6845, Australia; 3 Centre for Cardiovascular and Chronic Care, School of Nursing and Midwifery, Curtin University, Perth, Western Australia 6845, Australia; 4 Heart Foundation of Australia (WA), 334 Rokeby Road, Subiaco, Western Australia, 6008, Australia; 5 Royal Perth Hospital, GPO Box X2213, Perth, Western Australia 6001, Australia; 6 Derbarl Yerrigan Health Service, 156 Wittenoom Street, East Perth, Western Australia 6004, Australia; 7 Aboriginal Health Council of Western Australia, PO Box 8493, Stirling Street, Perth, Western Australia 6849, Australia

## Abstract

**Background:**

Cardiovascular disease is the major cause of premature death of Indigenous Australians, and despite evidence that cardiac rehabilitation (CR) and secondary prevention can reduce recurrent disease and deaths, CR uptake is suboptimal. The National Health and Medical Research Council (NHMRC) guidelines *Strengthening Cardiac Rehabilitation and Secondary Prevention for Aboriginal and Torres Strait Islander people*s, published in 2005, provide checklists for services to assist them to reduce the service gap for Indigenous people. This study describes health professionals' awareness, implementation, and perspectives of barriers to implementation of these guidelines based on semi-structured interviews conducted between November 2007 and June 2008 with health professionals involved in CR within mainstream health services in Western Australia (WA). Twenty-four health professionals from 17 services (10 rural, 7 metropolitan) listed in the WA Directory of CR services were interviewed.

**Results:**

The majority of respondents reported that they were unfamiliar with the NHMRC guidelines and as a consequence implementation of the recommendations was minimal and inconsistently applied. Respondents reported that they provided few in-patient CR-related services to Indigenous patients, services upon discharge were erratic, and they had few Indigenous-specific resources for patients. Issues relating to workforce, cultural competence, and service linkages emerged as having most impact on design and delivery of CR services for Indigenous people in WA.

**Conclusions:**

This study has demonstrated limited awareness and poor implementation in WA of the recommendations of the NHMRC *Strengthening Cardiac Rehabilitation and Secondary Prevention for Aboriginal and Torres Strait Islander Peoples: A Guide for Health Professionals*. The disproportionate burden of CVD morbidity and mortality among Indigenous Australians mandates urgent attention to this problem and alternative approaches to CR delivery. Dedicated resources and alternative approaches to CR delivery for Indigenous Australians are needed.

## Background

Cardiac rehabilitation (CR) programs are widely recommended for individuals with coronary heart disease (CHD) and widely endorsed in public health policy [1,2]. Despite the survival benefits of CR and improvements in quality of life, participation rates are low even among non-Indigenous Australians [3]. Aboriginal and Torres Strait Islander Australians are even less likely to participate in CR programs than non-Indigenous Australians, despite being more than twice as likely to die from cardiovascular disease (where the term 'Indigenous' occurs in this document it encompasses both Aboriginal and Torres Strait Islander people) [4]. Approximately 16% of all Indigenous Australians live in Western Australia (WA), and they comprise 3.8% of the total WA population. The Indigenous population is increasing at a higher than average rate of 2.5% per annum. Indigenous people are the least healthy of all Western Australians. The prevalence of chronic conditions is high and gaps persist between the health status of Indigenous and non-Indigenous people [5]. Indigenous people living in rural WA generally have higher rates of mortality and hospitalisation than those living in metropolitan regions.

While the incidence of coronary heart disease (CHD) has been declining over the last three decades in the general population, this trend has not occurred in Indigenous Australians, in spite of an enabling policy context. Cardiovascular disease (CVD) also occurs at a younger age, with rates in the 35-54 year age group four times that in Indigenous compared to non-Indigenous populations [6]. A previous audit by CR staff at the major tertiary hospital that receives referrals of Indigenous people from across WA, found few Indigenous patients ever attended outpatient CR and a tiny proportion returned for follow-up (personal communication, Narelle Wilson). Yet despite the burden of cardiovascular disease and the well-described problems in Indigenous Australians, few effective interventions are described. Community involvement, engagement of Aboriginal Health Workers (AHWs) and program delivery within a framework of cultural competence are thought to be important factors in effective service provision [7,8]. Cultural competence refers to a set of skills, attitudes and beliefs that enables an individual to work effectively in cross-cultural situations and is increasingly recognised in achieving access and equity to service provision [9].

Guideline development is an important step in driving practice change and promoting cultural awareness. Yet it is well-recognised that guidelines alone do not result in either practice or workplace culture and values changes. This study investigated practitioners' awareness and implementation of *Strengthening Cardiac Rehabilitation and Secondary Prevention for Aboriginal and Torres Strait Islander Peoples: A Guide for Health Professionals *[10] in CR services in WA. The Guidelines (key features are shown in Appendix 1) were developed using available evidence and a consultation process with key stakeholders. Through providing definitive information on services for Indigenous patients, this study aimed to help better plan CVD services for Indigenous Australians in WA. Although some Aboriginal Community Controlled Health services provide CR services, for the most part formalised programs are associated with post-hospital discharge programs. Therefore, this report focuses on mainstream health service CR programs.

Key to increasing participation of Indigenous people in these programs is the understanding of barriers and facilitators from their perspectives.

## Methods

The sampling frame for this study included public hospitals and community-based public CR services listed in the Directory of Western Australian Secondary Prevention Services [11]. Health professionals were recruited by a CR nurse who contacted rural and metropolitan health service organisations, initially by phone or e-mail, to explain the study. Semi-structured interviews were conducted between November 2007 and March 2008 by a CR nurse in conjunction with an Indigenous nurse whenever possible. The inclusion of an Indigenous nurse in the visits was to assist with assessment of the CR service in terms of its cultural safety and as a strategy for Indigenous research capacity building within the research. All interviews were conducted face-to-face to enable assessment of the atmosphere and how "Indigenous-friendly" the environment was in terms of supporting culturally safe care, as well as ensuring interviewees were fully engaged during the interview. Visits also enabled opportunities for providing information and education to participants as a form of reciprocity. The interviews were partially structured using quantifiable close-ended questions regarding awareness and implementation of the NHMRC *Guidelines *(Appendix 2), but also allowed for more in-depth discussions not fully captured in the question list regarding the experiences and perceptions of CR services for Indigenous people. All but three interviews were audio-recorded with permission of participants. Written notes were taken for those who declined recording. Transcripts and notes were analysed using thematic content analysis [12]. Frequencies of categorical responses to closed-ended questions were computed. Analysis involved open coding, in which the data was broken down into distinct units of meaning, or codes, according to participants' responses. The axial coding stage involved continuous comparisons of the codes with one another to discover links between the categories [12]. Related categories were combined and compared to new data. Steps were undertaken to maximise reflexivity and rigour through discussion within the research team and verification and clarification of themes emerging from the data [13]. Ethical Approval was obtained from the WA Aboriginal Health Information and Ethics Committee and the Curtin University Human Research Ethics Committee.

## Results

Several services listed in the Directory of WA Secondary Prevention Services were not interviewed, including those who had few or no CR patients/referrals (*n *= 4), serviced negligible numbers of Indigenous clients (*n *= 7, six of which were private services), or where staff were unavailable during data collection or failed to respond to invitations to participate (*n *= 3). Participating programs were in rural settings (*n *= 10, 59%) and the metropolitan area (*n *= 7, 41%). Twenty-four interviews were conducted with participants from 17 tertiary hospitals and community-based public CR services. Of the respondents who reported having received the *Guidelines *(n = 6, 25%), few could recall any specific elements or recommendations, nor did they report attempting to implement the approaches recommended in the resource. Findings related to Indigenous inpatients and outpatients are summarised below. The denominator for data reported below relates to participants in interviews (*n *= 24).

### Indigenous-specific awareness and services

A quarter of respondents reported having joint initiatives with an Aboriginal Medical Service (AMS), while nearly one-third reported faxed or telephoned patient referrals to/from an AMS. Fewer than half of respondents reported that their service has a system in place for identifying Indigenous status (*n *= 10; 41.7%). A minority (*n *= 2; 8.8%) reported having Indigenous community input into CR design and delivery.

### Services for Indigenous in-patients

Respondents explained that during a hospital stay, they usually did not visit Indigenous patients. Of those who do see Indigenous in-patients, 29% (*n *= 7) reported discussing with them the importance of CR. The majority of respondents did not have access to Indigenous-specific education resources on common CVD conditions, tests, interventions, and medications for patients during their hospital stay. However, a minority (*n *= 4; 17%) of programs reported providing culturally appropriate resources and had a buddy/mentoring scheme in place for Indigenous people hospitalised for a heart condition (*n *= 2; 8%).

### Post-discharge services for Indigenous patients

Only 8% (*n *= 2) of services reported being notified by tertiary hospitals when local Indigenous patients were discharged back to their area following a cardiac event, while a further 46% (*n *= 11) reported inconsistent notification. When referrals were obtained, 58% (*n *= 14) reported seeing Indigenous patients following discharge and of these, 62.5% (*n *= 15) reported talking about the importance of CR during this time and 37% (*n *= 9) had Indigenous-specific education materials. Only one post-discharge mentoring system was reported.

### Perceived barriers and facilitators to service access

Respondents identified the following barriers they were aware of that could prevent Indigenous people's access to services: family commitments and restrictions (*n *= 15, 62.5%); lack of awareness of available services (*n *= 14, 58%); lack of transport (*n *= 14, 58%); other health issues (*n *= 9, 38%); and financial constraints (*n *= 7, 29%). Many of the services offered CR only within very limited hours, compounding geographical barriers to access. Issues relating to workforce, cultural competence, and service linkages emerged as having most impact on design and delivery of CR services to Indigenous people in WA (Table 1). Verbatim quotes are provided in italics to illustrate themes.

**Table 1:** Themes and key issues identified by participants as having a major impact on design and delivery of CR to Indigenous people

Theme	Key issues
**Workforce**	• AHWs were described as being pivotal in engaging and maintaining relationships with Indigenous patients and their families.
	• AHWs are important in mentoring other health professionals by providing cultural insights into care of Indigenous people:
	"*One of the most important things in providing health services to Aboriginal people is to actually work with the people who have the cultural awareness, and that is the health workers, of course, Aboriginal people themselves...The people want services to be provided by their own people."*
	• Only 54% of respondents reported having access to Aboriginal and Torres Strait Islander staff which was a major impediment to engaging Indigenous people within mainstream services.
	• Existing AHWs faced limited infrastructure and support:
	*"They can't represent their community in that sort of environment, so they leave"*
	• High turnover of non-Indigenous staff impacted on initiative sustainability:
	*"I'm just relieving for J...". "...been here for three months" "...leaving in two weeks"*
**Cultural competence**	• Failure to appreciate reasons for poor participation:
	*"I don't know why they don't come*. "
	• Features of programs and services are not congruent with Indigenous clients' lifestyles, culture, commitments, and preferences:
	*"Maybe we could run an ATSI-specific class, because they have this huge shame factor when they are with other people and stuff that they don't like doing"*
**Linkages**	• Few systematic processes for identifying Indigenous people Inadequate communication and referral upon discharge:
	*"We don't know exactly what day they (Aboriginal patients discharged from hospital) are getting back. There are issues around the continuity of care even though we should get a discharge summary"*.
	• Disparate health information management systems between organizations
	• Lack of awareness of available services in different areas:
	*"It's probably our fault as there is not a good relationship between us and the cardiologists in Perth or the surrounding areas, because people might not think the service is available"*
	• Operating in isolation rather than with existing services

## Discussion

Despite the ready availability of NHMRC guidelines to all CR services in WA, the majority of respondents reported that they were unfamiliar with this document and as a consequence had not attempted to implement its recommendations. Although there were examples of good practice, we were unable to identify evidence of a systematic implementation strategy or outcome assessment strategy across WA. It is well-documented that guideline implementation is a complex and multifaceted process that needs to consider systems, patient and provider issues [14]. To effectively implement guidelines, a number of strategies should be undertaken underpinned by a targeted marketing and dissemination strategy. No one single strategy is adequate. Strategies such as involving consumers and professional groups, implementing technological solutions, as well as providing incentives have been shown to be successful [15-17]. Yet, this study makes evident that there are some entrenched challenges to service delivery that are unlikely to be solved by guideline development alone [18]. Workforce shortages and turnover, inferior and inadequate communication across care sectors and the barriers associated with rural and remote Australia remain a challenge. These issues are compounded by the socioeconomic and other issues facing Indigenous people in rural and remote Australia. This underscores the need to focus on structural reform to improve health outcomes for Indigenous Australians on a whole-of-community level. This involves providing safe communities, engagement of communities, and promotion of educational strategies [5].

Another important consideration in of the delivery of CR services is the interface between state and federal government initiatives [18]. Commonly, CR services are aligned with hospital services that are funded by state governments, whereas many community-based services are funded by the Australian Government. The study has highlighted several factors inhibiting sustainable funding models for CR in Indigenous people. There is a need for more integrated strategies in promoting cultural awareness and ensuring that these are integrated in service delivery. The lack of Indigenous staff as members of the health team was identified by many respondents as a major impediment to delivering culturally appropriate care. This study also identified the tensions between a standard approach to medical care and secondary prevention and the needs of Indigenous people, underscoring the importance of developing integrated models of care that are suitable to Indigenous problems [19]. The lack of awareness and consequent overlooking of involvement with Aboriginal Community Controlled Health Services among most respondents will likely limit the capacity to coordinate services; implementation of this needs higher level commitment and support. Further, the limited access to psychological and social services fails to address the complex interplay between physical and mental concerns in Indigenous health [20]. Limited transportation and family commitments remain key inhibitors to CR attendance as has also been noted in non-Indigenous people [21]. The potential to link Indigenous family and community education and interventions with follow-up and support for an index case was beyond the capacity of services, despite its attractiveness as a means of enhancing support to the patient and strengthening primary and secondary prevention in the wider Indigenous community. Efforts to strengthen the competencies of health staff to work with disadvantaged clients may help providers engage Indigenous people in CR, particularly if there is health management support for this as an important component of policy and practice [22].

When interpreting these study findings, consideration should be given to the method of sampling which may have reflected the view of health professionals who had not necessarily been working in that service over a long period. Further, although our informants were those most likely to know the situation within the CR service and at the patient-CR provider interface, there was no source data verification from health service management regarding the opinions expressed. In spite of these limitations, this study elucidated barriers and facilitators to guideline implementation and strategies to improve Indigenous Health in WA. In addition, it underscores the importance of following up guideline release with targeted implementation and evaluation strategies. Further, it emphasises the importance of policy diffusion from a state and federal level to a local service delivery context. Importantly, the views reported are the views of clinicians responsible for CR service implementation and guideline adherence in the real world. It is important to understand these views in improving services. This information provides a way forward for service planning and underscores an urgent need to invest in enabling health providers to provide culturally appropriate service models.

## Conclusions

Although evidence-based guidelines are integral for clinicians and supporting service reform, this study demonstrated sub-optimal awareness in WA of *Strengthening Cardiac Rehabilitation and Secondary Prevention for Aboriginal and Torres Strait Islander Peoples: A Guide for Health Professionals *among the professionals at whom they were targeted. Potentially limited endorsement and recognition at a local policy level have influenced this. Unsurprisingly, limited uptake of the recommendations was also identified. The disproportionate burden of CVD morbidity and mortality among Indigenous Australians, particularly following an acute cardiac event, requires urgent attention to ensure culturally appropriate and competent secondary prevention services. Alternative approaches to how CR services could be delivered are needed.

## Competing interests

The authors declare that they have no competing interests.

## Authors' contributions

SCT contributed to study design, securing funding, ethics approval, interpretation and writing, MLD assisted with analysis, interpretation and writing; JSS assisted with study design, data collection, stakeholder engagement and interpretation, KPT assisted with data collection and interpretation, LD assisted with conception, funding and stakeholder engagement, MA assisted with study conception and analysis, MMW assisted with stakeholder engagement, TGL assisted with study development and stakeholder engagement, PMD assisted with analysis, interpretation and writing. All authors approved the final manuscript.

## Appendix 1: Key Features for Successful Cardiac Rehabilitation in Indigenous Australians [10]

• Ensure cultural competency is integral to the core business of an organization and supported at all levels within the organization.

• Involve Aboriginal Health Workers and family members in the care of Indigenous Australians and develop flexible approaches to highlighting the merit of CR.

• Draw on existing CR and secondary prevention services as appropriate and engage with local community networks.

• Ensure community involvement at every level in planning and implementing CR, including the development of culturally appropriate resources.

• Develop and sustain partnerships between stakeholder agencies.

• Tailor CR approach according to the specific needs of Indigenous Australians and develop supportive policies and procedures.

• Develop specialist education resources for continuing professional development and support of all health professionals working in heart care, including Aboriginal Health Workers.

## Appendix 2: Topics guiding semi-structured interviews with cardiac rehabilitation providers

• Awareness of NHMRC guidelines [10]

• Degree of incorporation of recommendations into routine practice

• Barriers to implementation

• Aboriginal Health Worker involvement in care of Indigenous patients

• Collaboration and integration with existing service providers

• Cultural competency training

• Opportunities for continuing professional development

• Type, structure, content, organisation, coordination, staffing and funding of the service with specific relevance to Indigenous people
